# Use of Novel Biological Agent in Severe Ulcerative Colitis with Poor Response to Initial Therapy: A Case Report

**DOI:** 10.31729/jnma.7590

**Published:** 2022-09-30

**Authors:** Abashesh Bhandari, Bhupendra Kumar Basnet

**Affiliations:** 1 Department of Internal Medicine, Nepal Medical College and Teaching Hospital, Jorpati, Kathmandu, Nepal; 2 Department of Gastroenterology, Helping Hands Community Hospital, Chabahil, Kathmandu, Nepal

**Keywords:** *adalimumab*, *case reports*, *Nepal*, *therapy*, *ulcerative colitis*

## Abstract

Ulcerative colitis is a condition of chronic inflammation affecting the large intestine. The disease is typically characterized by periods of active inflammation alternating with periods of remission. For patients with moderate to severe ulcerative colitis, the first line for induction therapy options includes biologic agents (with or without an immunomodulator) or glucocorticoids. This case highlights the first successful case of ulcerative colitis treated with adalimumab with mesalamine and azathioprine. The patient was initially treated with mesalamine and azathioprine which did not result in significant clinical improvement but the addition of subcutaneous adalimumab showed a remarkable response with drastic improvement in just one year of treatment. Hence, adalimumab could be considered as an option in the management of severe cases in Nepal.

## INTRODUCTION

Ulcerative Colitis (UC) is a chronic immune-mediated inflammatory condition involving the large intestine causing significant morbidity and low mortality.^1^ Persistent disease activity despite the diagnosis and medical therapy can be expected in some patients and few present with rapid-onset progressive type colitis or fulminant disease.^2,3^ When therapy with conventional agents fails in such cases, an approach with novel treatment could be needed. Here, we report the first case of ulcerative colitis being successfully treated with adalimumab along with mesalamine and azathioprine with remarkable improvement in the one-year duration of treatment and to the best of our knowledge, such cases has not been reported in Nepal.

## CASE REPORT

A 37-years-old male patient presented with a prolonged history of per-rectal bleeding and lower abdominal pain which was insidious, non-radiating, intermittent, dull aching type with no apparent aggravating or relieving factors. He also had an increased frequency of defecation, which he reported to occur around 8 times per day. His past history, family history, and psychosocial history were insignificant. On examination, the patient had tachycardia. Other findings on examination were unremarkable.

On laboratory workup, the patient had increased total leukocyte count (TLC) (13000/cumm) along with increased neutrophils (88%) and lymphocytes (10%). Also, the erythrocyte sedimentation rate was 45 mm in the first hour. His stool microscopic examination revealed plenty of pus cells per high power field (HPF), red blood cells around 10-12 per high power field (HPF), and cysts of *Entamoeba histolytica.* However, his blood hemoglobin level was 14.2 gm/dl.

Concerning this, the patient underwent colonoscopy and biopsy which showed reports consistent with the diagnosis of ulcerative colitis with pancolitis with pseudo-polyp formation.

The Computed Tomography (CT) scan of the abdomen showed findings consistent with diffuse colitis. Based on the clinical findings and the reports, the patient's severity was classified as severe ulcerative colitis as per the True Love and Witts score. After this diagnosis, the patient was then started on an enema with mesalamine 4.8 gm per day.

One month later, the present came for follow-up when his symptoms were still persistent. After that, azathioprine 50 mg twice daily was started along with mesalamine tablets. Further, after 3 months since the initial presentation, the patient landed in the emergency department with an increase in the severity of the per rectal bleeding, lower abdominal pain, and abdominal distension. Workup on that day suggested an acute flare-up of ulcerative colitis with toxic megacolon. He was then admitted and managed in the intensive care unit (ICU), but his bleeding and pain persisted.

The patient was then started on intravenous adalimumab 160 mg subcutaneously along with mesalamine and azathioprine as per the ulcerative colitis protocol. The patient then remarkably responded to the given management and showed symptomatic improvement with no apparent side effects. The patient was then followed up after six months when the colonoscopy findings had also improved. The colitis extended to descending colon and the severity of the erythema and edema had also reduced. The treatment was continued to complete a one-year duration after which the patient was symptom-free with improved colonoscopy findings. There were no apparent adverse or unanticipated events following the treatment.

## DISCUSSION

Ulcerative colitis (UC) is a chronic inflammatory condition of the large intestine characterized by periods of active inflammation alternating with periods of remission.^2,3^ The severity can be classified using the True Love and Witts score.^4^ Frequency of bowel movement, per rectal bleeding, urgency, abdominal pain, cramping, and weight loss, as well as extraintestinal manifestations, are imperative in the diagnosis and severity stratification of UC. Also, potential precipitants such as recent smoking cessation,^5^ nonsteroidal anti-inflammatory drugs (NSAID) use,^6,7^ and enteric infections need to be identified.^8^

Markers such as C-reactive protein (CRP) and erythrocyte sedimentation rate (ESR)^9^ often correlate with the endoscopic severity of the disease^10^ and have a prognostic significance.^11^ Measurement of hemoglobin and serum albumin levels at diagnosis can help assess disease severity and prognosis.^4^

Our case reports a condition of pancolitis presenting with increased frequency of bowel movements, per rectal bleeding, abdominal pain, tachycardia which on workup revealed raised ESR, raised TLC and stool examination showed plenty of pus cells per high power field (HPF), red blood cells around 10-12 per HPF, and cysts of *Entamoeba histolytica.* However, his blood hemoglobin level was normal. Based on these features, a diagnosis of severe ulcerative colitis was made.

For the management of extensive colitis, a combination of an oral 5-AminoSalicylic Acid (ASA) with rectal mesalamine in high doses (>3 gm daily) is given in mild to moderate cases of UC.^12^ For patients with moderate to severe UC, the first line for induction therapy options include biologic agents (with or without an immunomodulator [eg. azathioprine, methotrexate, etc.]) or glucocorticoids. The biologic agents include infliximab, adalimumab (preferred in patients who are compliant with self-injections), vedolizumab (preferred in patients who are ≥65 years old or who have a history of recent infection, etc.^13,14^

Our case was of severe pancolitis which was managed initially with mesalamine and azathioprine which did not result in significant clinical improvement. Therefore, he was then started on subcutaneous adalimumab after which remarkable response was seen with the disappearance of the symptoms and improvement of the endoscopic picture. Adalimumab has not been used for the treatment of ulcerative colitis in Nepal to date to the best of our knowledge and our case's successful outcome with this therapy remains the key strength. The patient was highly satisfied and appreciative of the given treatment.

Hence, adalimumab could be a worthwhile method of treatment and could be considered as an option in the management of severe UC in Nepal, provided the patient will be compliant with subcutaneous injections. However, further controlled studies with a larger number of patients need to be performed in the Nepalese population to establish this.
